# Acetonitrile Adducts of Tranexamic Acid as Sensitive Ions for Quantification at Residue Levels in Human Plasma by UHPLC-MS/MS

**DOI:** 10.3390/ph14121205

**Published:** 2021-11-23

**Authors:** Eduarda M. P. Silva, Luisa Barreiros, Sara R. Fernandes, Paula Sá, João P. Prates Ramalho, Marcela A. Segundo

**Affiliations:** 1 TOXRUN—Toxicology Research Unit, University Institute of Health Sciences, CESPU, CRL, 4585-116 Gandra, Portugal; 2 LAQV, REQUIMTE, Department of Chemical Sciences, Faculty of Pharmacy, University of Porto, Rua de Jorge Viterbo Ferreira 228, 4050-313 Porto, Portugal; saraferns@sapo.pt (S.R.F.); msegundo@ff.up.pt (M.A.S.); 3 School of Health, Polytechnic Institute of Porto, Rua Dr. António Bernardino de Almeida 400, 4200-072 Porto, Portugal; 4 Centro Hospitalar Universitário do Porto, Largo Prof. Abel Salazar, 4099-001 Porto, Portugal; paulalexsa@gmail.com; 5 Department of Chemistry, School of Science and Technology, University of Évora, Rua Romão Ramalho 59, 7000-671 Évora, Portugal; jpcar@uevora.pt; 6 LAQV, REQUIMTE, University of Évora, Rua Romão Ramalho 59, 7000-671 Évora, Portugal; 7 Hercules Laboratory, University of Évora, Palácio do Vimioso, Largo Marquês de Marialva 8, 7000-809 Évora, Portugal

**Keywords:** electrospray ionization, collision-induced dissociation, fragmentation, solvent adducts, analyte quantification

## Abstract

The quantitative analysis of pharmaceuticals in biomatrices by liquid chromatography coupled with electrospray ionization tandem mass spectrometry (LC-ESI-MS/MS) is often hampered by adduct formation. The use of the molecular ion resulting from solvent adducts for quantification is uncommon, even if formed in high abundance. In this work, we propose the use of a protonated acetonitrile adduct for the quantitative analysis of tranexamic acid (TXA) by LC-MS/MS. The high abundance of the protonated acetonitrile adduct [M + ACN + H]^+^ was found to be independent of source-dependent parameters and mobile phase composition. The results obtained for TXA analysis in clinical samples were comparable for both [M + ACN + H]^+^ and [M + H]^+^, and no statistically significant differences were observed. The relative stability and structure of the [M + ACN + H]^+^ ions were also studied by analyzing probable structures from an energetic point of view and by quantum chemical calculations. These findings, and the studied fragmentation pathways, allowed the definition of an acetimidium structure as the best ion to describe the observed acetonitrile protonated adduct of TXA.

## 1. Introduction

The requirements for the determination of target compounds at sub-microgram per milliliter levels in a variety of matrices challenges every day analytical chemists towards the development of sensitive and quantitative methods. One of the most widely used techniques for the quantification of biologically active compounds is liquid chromatography coupled to tandem mass spectrometry (LC-MS/MS). Electrospray ionization (ESI), on the other hand, is the most frequently used mode of ionization connected to LC-MS. Concerning the quantitative analysis of pharmaceuticals in biomatrices with ESI-LC-MS/MS, the most used ions are [M + H]^+^ or [M + H]^−^ acquired from positive (ESI+) or negative (ESI-) ion mode, respectively. Nonetheless, these ions are not always the most abundantly formed and quantitative analysis is often impaired by adduct formation through the decrease in abundance of these *pseudo*-molecular species [1]. Moreover, most studies are frequently concerned with monitoring only ions that have high signal intensities not considering structural interpretation important to evaluate the consistency with the chemical structure of the analyte under study and avoid incorrect assignments. This careful analysis is of extreme importance because not only it provides accurate quantification but also qualitative identification and confirmation, essential for the correct analysis of biological phenomena [2,3,4,5].

Several features can affect the ESI process, namely the composition and flow rate of the mobile phase, the use and concentration of additives, pH, and temperature [6]. Hence, depending on these parameters and analyte nature, different types of ions may be formed. Formation of ion species with alkali metal cations (e.g., [M + Na]^+^ and [M + K]^+^) instead of proton addition is often observed in the positive ion mode of ESI leading to adducts of difficult fragmentation [7,8,9,10,11]. These adducts are formed when analytes in solution hold inherent dipoles that interact with these cations available from mobile phase additives, solvent impurities, and glassware, being difficult to control or eliminate. This unpredictability delivers a process with a variable response creating a source of non-reproducibility of mass signals. Nevertheless, lithium adducts, for example, have been successfully used as precursor ions for sensitive and rapid quantification of natural products in biological samples [12,13]. Sodium versus protonated adduct formation efficiency and sensitivity in ESI-MS have also been evaluated by using different mobile phase additives ranging from a pH of 2.1 to 10.7 [14]. Seventeen compounds with four different possible sites for the formation of adducts were studied in the ESI+ mode. The authors concluded that the use of mobile phase additives was beneficial towards repeatability and that oxalic acid, for example, was recommended for protonated molecule measurements, while acetic acid or formic acid additives were suitable for sodium adduct monitoring.

Acetonitrile (ACN) and methanol, two of the most frequently used solvents for LC-MS, are also known to form abundant adducts under electrospray ionization [15,16,17,18,19,20]. The use of these solvent adducts for analytical purposes must be carefully considered and their linear behavior versus analyte concentration needs to be assessed. Difficulties arise when multiple reaction monitoring (MRM) is considered for quantitative LC-MS/MS methodologies. This is mainly due to low signal and adduct formation reproducibility, since adduct formation is sensitive to ionization conditions and prone to decomposition, and complexity of the corresponding mass spectra. In addition, a spectrum with multiple ions of the target compound makes it difficult the selection of the most suitable one for MRM. The summation of all adduct ions can be a possibility. However, the response factor for all adducts might not be equal leading to unreliable results. Nevertheless, the use of isotopically labeled internal standards and relative measurements of adduct formation can compensate for variations in adduct formation. Unfortunately, these types of compounds are not always available and represent an additional cost for analysis implementation.

**Scheme 1:**
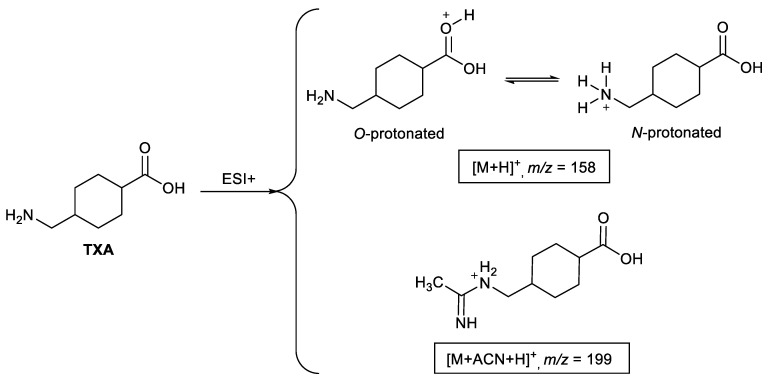
Structure of the TXA-ACN protonated adduct formed by positive-mode electrospray ionization and the two possible protonation sites of tranexamic acid.

Several methods have been proposed for the quantification of the antifibrinolytic agent tranexamic acid (TXA, Scheme 1) in biological fluids including human plasma and serum [21]. Reported methods based on LC-MS/MS describe the use of *m*/*z* transitions 158.2 > 95.2 and 158.2 > 140.7 for selected reaction monitoring (SRM) allowing detection limits ranging from 0.01 to 0.5 μg mL^−1^ [22,23,24,25,26,27,28,29]. These transitions, and others used for identity purposes, are all the result of the fragmentation of the protonated *pseudo*-molecular ion [M + H]^+^.

In the course of our studies related to the development of an LC-MS/MS method to quantify TXA in human plasma, a protonated acetonitrile adduct ([M + ACN + H]^+^) was observed as the most abundant ion formed under electrospray ionization in the positive mode (Scheme 1) [30]. Due to the stability displayed by this TXA-ACN protonated adduct ion under the reported conditions, its potential application was recognized for quantification purposes allowing to achieve higher sensitivity. Therefore, having in mind this possibility, the aim of the present work was to understand the formation and stability of the main TXA-ACN protonated adduct ion in LC-ESI-MS/MS analysis. Moreover, the significance of the observed adduct signals for the detection and quantification of TXA in biological samples was evaluated.

## 2. Results and Discussion

### 2.1. Ionization Studies

**Figure 1:**
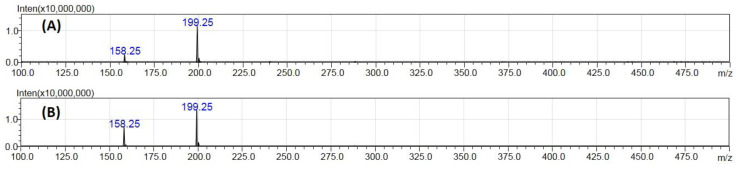
ESI-MS scan spectra obtained for TXA using (**A**) acetonitrile-aqueous ammonium bicarbonate (pH 7.4; 10 mM) (80:20, *v*/*v*) and (**B**) acetonitrile-aqueous ammonium bicarbonate (pH 7.4; 10 mM) (50:50, *v*/*v*) as mobile phase.

Positive and negative-ion ESI-MS studies of a tranexamic acid (TXA) standard solution at 10 μg mL^−1^ in water were performed in the scan mode by flow injection analysis into the ion source at a flow rate of 0.1 mL min^−1^ (Figure S1). A mixture of acetonitrile-aqueous ammonium bicarbonate (pH 7.4; 10 mM) (80:20, *v*/*v*) was used as mobile phase. At low cone voltages, the TXA molecule is more easily ionizable, as expected, in the positive mode. Mass spectra demonstrated the intact cations [M + H]^+^ and [M + ACN + H]^+^ as two abundant peaks corresponding to monoisotopic masses of 158.25 and 199.30 Da. The base peak of the mass spectra corresponded to the [M + ACN + H]^+^ with a 1.2 × 10^7^ ion current (Figure 1A). In these conditions, the relative abundance of [M + H]^+^ ion was 21.4 ± 1.5%. In the negative mode, TXA was ionized into the negatively charged ion [M-H]^−^ at *m*/*z* 156.25 but the signal intensity was reduced and no acetonitrile adduct ions were observed (Figure S1). Therefore, only the positive mode was considered for further studies.

Besides the protonated adduct [M + ACN + H]^+^, other adduct ions could be formed with acetonitrile in the positive mode, such as [2M + ACN + H]^+^ or [M + 2ACN + H]^+^ [14] corresponding to *m*/*z* 356 or *m*/*z* 240, respectively. However, as depicted in the Scan MS spectra presented in Figure 1, the protonated ion with one molecule of acetonitrile at *m*/*z* 199 was the only adduct formed and therefore the only one considered in the present work.

Several attempts were made to selectively maximize the ionization efficiency of the target *pseudo*-molecular ion [M + H]^+^ and reduce competitive adduct formation, which is the most generally applied approach. Initially, source-dependent parameters such as desolvation line temperature, heat block temperature, interface voltage, nebulizing gas flow rate and drying gas flow rate were studied and their effect in the response observed for [M + H]^+^ and [M + ACN + H]^+^ was evaluated. The influence of ESI temperature was not assessed as the ESI probe is not heated in the used MS equipment. Heat block temperature (350–500 °C) and interface voltage (3.5–5 kV) had no significant influence in the ratio [M + H]^+^/[M + ACN + H]^+^. On the other hand, the increase of nebulizing gas flow rate (1–3 L min^−1^) resulted in higher total signal intensities. However, this increment in nebulizing gas had a negative effect in the formation of *pseudo*-molecular ion [M + H]^+^ and for the maximum tested values the ion at *m*/*z* 158.25 was not detected. Desolvation line temperature (200–280 °C) and drying gas flow rate (0–18 L min^−1^) were the parameters exhibiting most influence in adduct detection. Indeed, the use of the maximum values of these parameters favored the desolvation of the adduct ion, reducing its intensity and enhancing the signal of the target ion [M + H]^+^. Nevertheless, none of the tested conditions permitted to completely abolish the formation of solvent adduct ions that continued to constitute the base peak of the mass spectra. According to these studies, the final selected conditions were as follows: nebulizing gas flow rate at 1.5 L min^−1^, drying gas flow rate at 18.0 L min^−1^, desolvation line temperature at 280 °C, heat block temperature at 400 °C, and interface voltage automatically adjusted according to the equipment tuning file.

The effect of mobile phase composition in the ratio [M + H]^+^/[M + ACN + H]^+^ was also assessed when using flow injection analysis and chromatography separation (Table 1). Considering the flow injection analysis, the percentage of acetonitrile in the mobile phase was varied between 50 and 80% (*v*/*v*) and it was observed that lower organic solvent contents favoured the ionization of TXA *pseudo*-molecular ion [M + H]^+^. In fact, the ratio [M + H]^+^/[M + ACN + H]^+^ increased significantly (*p* < 0.05) from 21.4 ± 1.5% with 80% (*v*/*v*) ACN to 54.7 ± 4.2% with 50% (*v*/*v*) ACN (Table 1). Lower organic solvent percentages corresponded to less ACN reaching the ionization source and, therefore, diminished the probability for formation of adduct ions.

The same test was performed with hydrophilic interaction-based chromatographic separation of TXA (BEH Amide column, 50 × 2.1 mm, 1.7 µm) prior to MS detection. In this case, the same effect of organic solvent content in ionization was observed but the resulting ratio [M + H]^+^/[M + ACN + H]^+^ values were significantly lower (|t|*_calculated_* > t*_tabulated_* (*p* = 0.05) = 2.57) in comparison to the values obtained using flow injection analysis raising from as low as 10.5 ± 0.1% with 80% (*v*/*v*) ACN to 33.8 ± 0.1% with 50% (*v*/*v*) ACN (Table 1). In comparison with flow injection analysis, reduced analyte dispersion is observed when chromatographic separation is performed before MS detection, which probably minimizes the efficiency of [M + ACN + H]^+^ adduct desolvation and thus permits to increase its relative intensity. Despite the highest analytical signal for [M + H]^+^ obtained with 50% (*v*/*v*) ACN (Figure 1B), TXA was not retained in the stationary phase in these conditions. Therefore, having in mind the development of an LC-MS/MS method for analysis of plasma samples [30], organic solvent percentages lower than 50% (*v*/*v*) were not considered.

The influence of different mobile phase additives was also studied through the addition of formic acid 0.1% (*v*/*v*) instead of aqueous ammonium bicarbonate (pH 7.4; 10 mM). The use of formic acid did not lead to any improvement of [M + H]^+^ versus [M + ACN + H]^+^ formation. Moreover, TXA was not retained in the stationary phase when an acidic mobile phase was employed for separation and thus the use of formic acid was not further investigated.

The relative intensities of TXA *pseudo*-molecular ion and acetonitrile adduct also changed with mobile phase flow rate. When the flow rate was increased from 0.1 to 0.2 and 0.4 mL min^−1^ using a mobile phase containing 80% (*v*/*v*) ACN, the relative intensity of *m*/*z* 158.25 was significantly reduced from 10.5 ± 0.1% to 3.0 ± 0.2% and 0.7 ± 0.1%, respectively. Lower flow rates imply that the column eluate enters the ion source more slowly and therefore the time available to desolvate adduct ions is extended, permitting to maximize the ratio [M + H]^+^/[M + ACN + H]^+^.

It is important to note that the ionization behavior of TXA and the TXA-ACN protonated adduct formation was only evaluated using an ESI interface in one instrument (Shimadzu triple quadrupole) and one source design (orthogonal). As reported by Mortier et al. [11], differences can be observed between different ionization sources and geometry in responding to additive adducts. Moreover, different ionization sources, geometries, and detectors will present different sensitivities, yielding enhanced or reduced limit of detection (LOD) and limit of quantification (LOQ) [1]. Thus, the permutability of the developed method, including sensitivity, LOD and LOQ values, should be assessed if one requires to determine TXA using a different instrument.

### 2.2. Fragmentation Mechanisms and DFT Calculations

**Figure 2:**
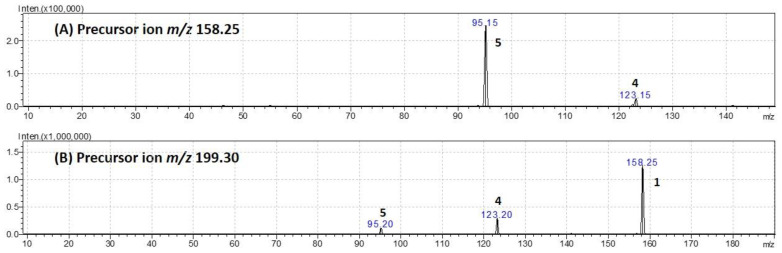
Product ion spectra (CID-MS/MS) obtained at CE of −10 V of the selected precursor ions (**A**) [M + H]^+^ at *m*/*z* 158.25 and (**B**) [M + ACN + H]^+^ at *m*/*z* 199.30.

Protonated TXA molecules, [M + H]^+^, generated by ESI+ can exist with the proton residing on either the amine nitrogen or the carboxylic acid oxygen as shown in Scheme 1, with the *N*-protonated form being 22.8 kcal mol^−1^ in gas phase and 35.3 kcal mol^−1^ in solution lower in energy than the *O*-protonated form. This result suggests the amine as the most suitable protonation site and the *N*-protonated form as the most stable cation when compared with the *O*-protonated form. However, the protonated ion is not the species formed with the highest relative abundance under the LC-MS described conditions. The base peak in the spectrum is displayed at *m*/*z* 199.30 corresponding to the [M + ACN + H]^+^ ion resulting from the addition of a molecule of acetonitrile to TXA. In order to study the product ions formed for both the [M + H]^+^ and [M + ACN + H]^+^ ions, the collision-induced dissociation mass spectra (CID-MS/MS) were obtained. For these experiments, the Q1 and Q3 were set to select distinctively precursor and product ions, using argon in Q2 for collisional induced dissociations. Collision energy (CE) values between −10 and −50 V were optimized through the mass spectrometer software to maximize the signal corresponding to each ion for quantification purposes. In Figure 2, the CID-MS/MS spectra of the [M + H]^+^ and [M + ACN + H]^+^ ions of TXA are shown for a CE of −10 V.

Analysis of the fragmentation behavior of [TXA + ACN + H]^+^ revealed a similar pattern to the observed for [M + H]^+^ (Figure 2). The adduct delivers only three product ions at *m*/*z* 158.25, 123.20, and 95.20 (Figure 2B) with the last two being common to the MS^2^ spectrum of the protonated *pseudo*-molecular ion (Figure 2A). For higher collision energy values, and because, in this case, product ions retain higher levels of energy, further decomposition occurs leading to fragmentation of the cyclohexane ring in TXA (data not shown).

**Scheme 2:**
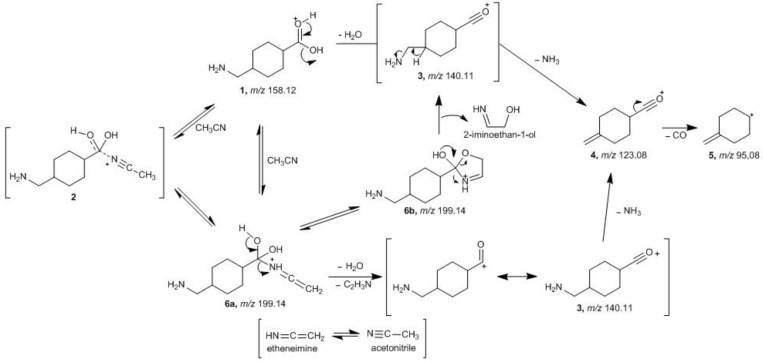
Gas-phase formation of [M + H]^+^ and [M + ACN + H]^+^ and its fragmentation pathway.

Considering the protonation in TXA mechanistically, only the *O*-protonated [M + H]^+^ ion can lead to the formation of the acetonitrile adduct (**structure 2**, Scheme 2). The high stability of the ion at m/z 199.30 implies the formation of a stable complex between the [M + H]^+^ ion and the acetonitrile molecule. To gain a better understanding of this nucleophile attack of the acetonitrile nitrogen (Ritter-type attack [31]), the relative stability of the [M + ACN + H]^+^ adduct (**2**) has been calculated.

**Figure 3:**
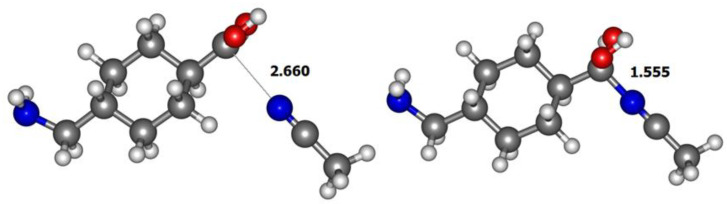
Optimized geometries M06-2X/6-31+G(d,p) level of the [M + ACN + H]^+^ adduct (**2**) in the gas phase (**left**) and in acetonitrile (**right**).

The calculation for the adduct in the gas phase (Figure 3) reveals the formation of a stable complex with relative energy of −17.3 kcal mol^−1^, when compared with the separated [M + H]^+^ ion and the acetonitrile molecule, and a quite elongated distance between the carbon of the carbonyl group and the nitrogen of the acetonitrile molecule of 2.67 Å. The complexation energy has also been calculated at the mp2/6-311+(2d,p) level as −18.61 kcal mol^−1^ result. When acetonitrile solvent is taken into account a complex with a distance between the carbon of the carbonyl group and the nitrogen of the acetonitrile molecule of 1.56 Å was found. For this complex, however, the relative energy when compared with the isolated solvated [M + H]^+^ and acetonitrile is 4.0 kcal mol^−1^ at M06-2X/6-31+G(d,p) level and 4.2 kcal mol^−1^ at MP2/6-311+G(2d,p) theory level.

The [M + ACN + H]^+^ adduct (**2**) can also suffer conversion to other stable structures where a covalent bond is generated, after a prototropic tautomerization pathway leading to the proposed *N*-vinylidenemethanaminium **6a** and/or the dihydrooxazolium **6b**.

The formation of the protonated acetonitrile complex **2**, as proposed in the fragmentation pathway described in Scheme 2, is reversible leading to the formation with a high relative abundance (74.9% RA) of product ion **1** (Figure 2B, *m*/*z* 158.25), by loss of a neutral fragment with a mass equivalent to a molecule of acetonitrile (41 Da). The formation of the product ion **4** (*m*/*z* 123.20, 17.8% RA) is produced by loss of 35 Da by concomitant or sequential elimination of H_2_O plus NH_3_ by proton-transfer-driven fragmentations, assuming the charge to be located on the oxygen atom of the carbonyl group of the carboxylic moiety. The loss of 28 Da, corresponding to the cleavage of the carbonyl group (CO), leads to the formation of ion **5** with low relative abundance (7.3% RA) at *m*/*z* 95.20.

**Figure 4:**
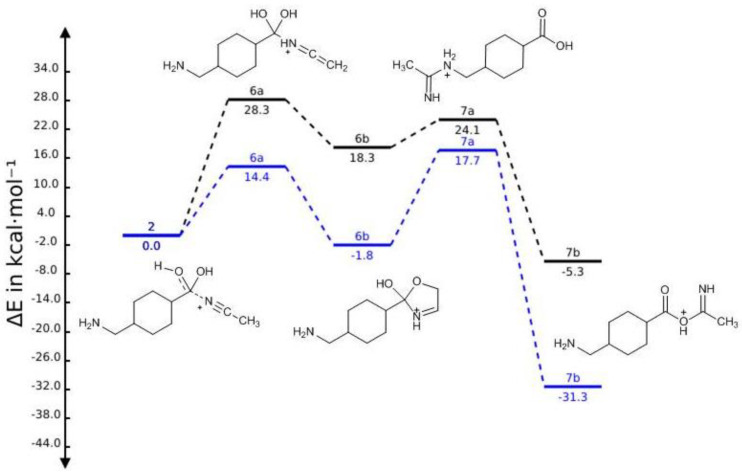
Calculated energies of the [M + ACN + H]^+^ species in the gas phase (black) and in the solvent environment (blue) relative to the energy of adduct **2**.

There are two other possible fragmentation pathways contributing directly towards the formation of product ion **3** (Scheme 2). Concerning adduct **6a**, the charge is considered to be located on the protonated nitrogen atom of the added ACN portion, and simultaneous elimination of H_2_O and etheneimine (59 Da) results in the formation of product ion **3** via a resonance structure comprising an acylium ion. Although product ion **3** is not observed in the spectrum, the fragmentation pathway leading to product ion **4** necessarily involves the formation of this intermediate and subsequent loss of NH_3_ (17 Da). Regarding adduct **6b**, the cyclization reaction occurs by attack of the hydroxyl group onto the ethenimine β-carbon of structure **6a**. Typically the β-carbon of ketenimines has a ‘carbanion’ character reacting with electrophiles at this position, rather than at nitrogen [32]. Collisional activation leads to opening of the oxazolium ring to give the acylium ion **3** and the neutral 2-iminoethan-1-ol (59 Da). Regarding the relative stabilities of the possible resulting species (Figure 4) considering these fragmentation pathways (Scheme 2) adduct **2** results to be the most stable species when compared with adduct **6a** in the gas phase and in the solvent environment while, when considering adduct **6b**, this is the energetically more stable ion in the solvent environment.

**Scheme 3:**
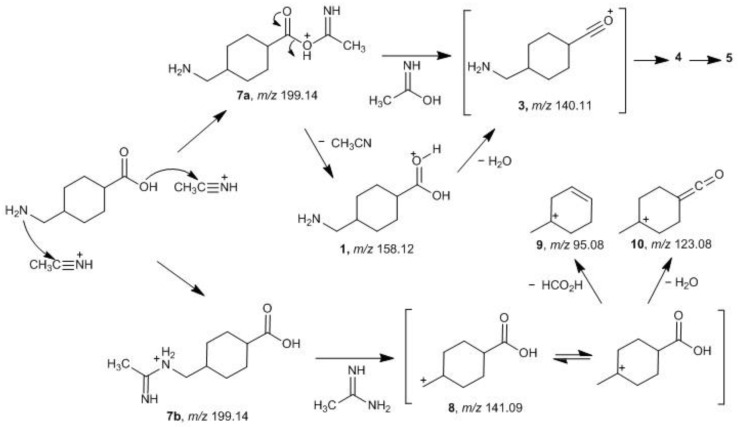
Gas-phase reaction of TXA with ethylnitrilium ion producing acetonitrile derivatives **7a** and **7b**.

**Figure 5:**
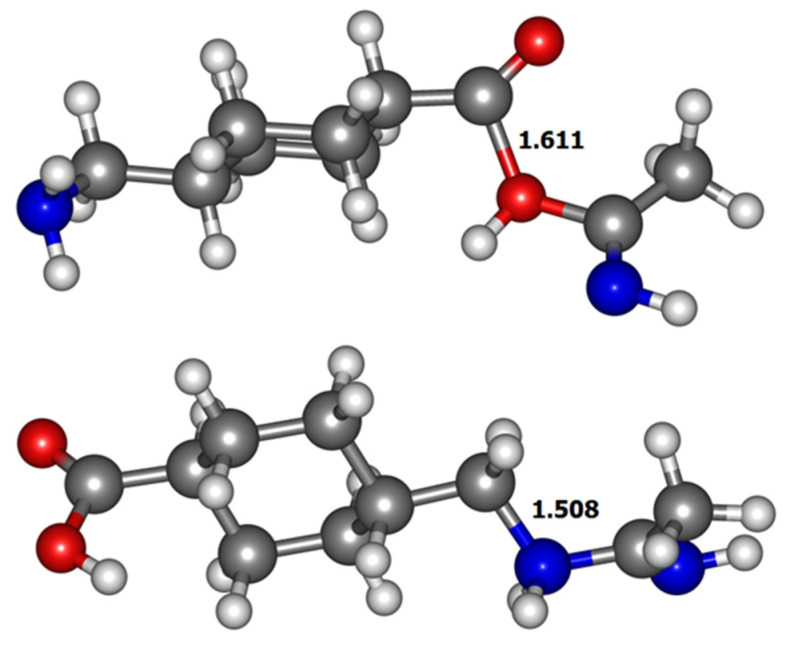
Optimized structures at M06-2X/6-31+G(d,p) level of the adducts **7a** (**top**) and **7b** (**bottom**) in acetonitrile environment with the length of the elongated bonds.

The formation of the TXA-ACN adduct can also be envisioned considering the reaction of the molecule with the protonated acetonitrile. Both the carboxylic acid and the amine functional groups can attack the electro-deficient carbon atom of the protonated acetonitrile leading to the formation of adducts **7a** and/or **7b** (Scheme 3). The ethylnitrilium ion can be generated under the conditions of ESI when ACN is used as a component of the LC-MS/MS mobile phase and participates in gas-phase reactions [33]. Considering the formed adduct **7a**, it can then rearrange to the corresponding *pseudo*-molecular ion **1** by loss of a molecule of acetonitrile and further fragmentation results in the formation of product ions **4** and **5** as described above. Cleavage of the C-O bond in structure **7a** with the resulting loss of an acetimidic acid molecule leads to the formation of the acylium ion **3** (Scheme 3). Furthermore, the optimized structure of this adduct (Figure 5) presents a significantly elongated bond, from 1.357 Å in the neutral compound to 1.611 Å in the protonated ion, between the carbonyl carbon and the protonated oxygen, prone to cleavage that results in the product ion **3**. It is known that fragmentation that occurs by simple cleavage, without rearrangements, is related to bond length increasing resulting from protonation [34]. On the other hand, the adduct ion **7b**, formed through the nucleophilic attack of the amine to the ethylnitrilium ion, can undergo the cleavage of the acetimidamide moiety affording the fragmentation ion **8** that yields, subsequently, by loss of formic acid that decomposes to H_2_O plus CO, a fragment ion of *m*/*z* 95.08. This is consistent with an elongation, and consequent weakening, from 1.452 Å in the neutral compound to 1.508 Å in the protonated ion **7b**, of the bond between the protonated nitrogen and the methylene group of TXA observed in the calculated structures.

From the adducts considered in both fragmentation pathways described, **7b** emerges as being the most energetically favorable structure of all considered [M + ACN + H]^+^ adducts species (Figure 4). Regarding the Gibbs energy values of the different adducts (data not shown), a similar picture emerges suggesting adduct **7b** as the thermodynamically more stable.

The performed theoretical calculations are in accordance with the experimental observations and suggest that acetonitrile adducts of TXA are not preformed in solution but formed only in the charged nanodroplets produced in ESI or in the gas phase [14]. In order to examine the possible formation of the protonated acetonitrile adduct in solution, the ^1^H NMR spectra of TXA were acquired in a deuterated acetonitrile/water mixture (75:25, *v*/*v*) and in this solvent mixture using a non-hydrogenated additive sodium carbonate, reproducing the same pH conditions used in the mobile phase (Figure S2). Tranexamic acid showed low solubility in the quoted deuterated solvents. Nevertheless, it was possible from those experiments to conclude the non-formation of the protonated acetonitrile because, for example, the expected methyl singlet in the aliphatic proton region observed for the TXA-ACN protonated adduct was absent. Moreover, all the observed signals correlated well with previously reported data [35,36].

### 2.3. Application of the Acetonitrile Adduct for TXA Quantification in Plasma Samples

To assess the feasibility of applying the acetonitrile adduct for quantification of TXA, standards and samples were analyzed by the UHPLC-MS/MS method previously described by this research group [30] but instead of monitoring product ions resulting from the fragmentation of the *pseudo*-molecular ion at *m*/*z* 158.25, product ions resulting from the fragmentation of the adduct ion at *m*/*z* 199.30 were used for quantification and identity confirmation.

Targeting the analysis of plasma samples, the acetonitrile percentage of mobile phase in the developed method was fixed at 75% (*v*/*v*) as a compromise between analysis time and an efficient separation of TXA from matrix components eluting within the void volume. In these conditions, the ratio [M + H]^+^/[M + ACN + H]^+^ was 19.8 ± 3.3%, i.e., the adduct ion represented approximately 80% of the total ion current.

The performance of the method employing the acetonitrile adduct for quantification was evaluated through the determination of several analytical figures of merit, namely linearity, limits of detection (LOD) and quantification (LOQ), precision and accuracy, recovery, and matrix effect (Table 2). Moreover, a comparison was performed with the values obtained when the *pseudo*-molecular ion of TXA was used for quantification.

Regression data analysis was performed to establish calibration curves for peak area ratio (TXA/TXA-IS) versus TXA concentration. The calibration curves were linear and reproducible between 30 and 600 ng mL^−1^ TXA (corresponding to 180–3600 ng mL^−1^ in plasma), with correlation coefficient *r^2^* ≥ 0.9983. As depicted in Table 2, the slope and *y*-intercept of these calibration curves were similar (*p* < 0.05) to the values previously obtained using the *pseudo*-molecular [M + H]^+^ ion for quantification. As calibration curves were constructed considering peak area ratios (TXA/TXA-IS), this result was expected and suggests the suitability of the adduct ion-based strategy for TXA quantification. Furthermore, back-calculated concentrations were within the recommended limits of ±15% of the nominal value (results not shown) [37].

The LOD and LOQ were estimated by the signal-to-noise ratio and the obtained values were, respectively, 3 and 6 ng mL^−1^ in plasma samples. When these values were compared to those previously obtained (Table 2) [30], there was a clear improvement on the LOD and LOQ (ca. 6x), evidencing the potential of adduct ion-based approach to detect and quantify TXA at trace levels. The achievement of lower LOD and LOQ values may be attributed to a reduced noise level and higher signal intensity values when monitoring the acetonitrile adduct.

The intra- and inter-day precision and accuracy values (Table 2) were acceptable, i.e., the precision, represented as CV, did not exceed 15% and the accuracy range was between 85 and 115% [37], indicating that adduct ion quantification yields reliable results.

Mean recovery values of 98.8% (CV = 0.5%) and 100.6% (CV = 1.0%) were attained for the target analyte TXA and the internal standard TXA-IS, respectively (Table 2). In accordance with the results observed previously with [M + H]^+^, extraction efficiency proved to be consistent and precise when adduct [M + ACN + H]^+^ ion-based quantification was performed. Moreover, the application of the statistical *t*-test to compare the mean recovery values obtained by the two quantification strategies evidenced no significant differences between the mean TXA recovery values obtained by the two methods (|t|*_calculated_* < t*_tabulated_* (*p* = 0.05) = 4.30). On the other hand, the recovery values attained for TXA-IS were significantly higher (*p* < 0.05) using the adduct-based strategy.

The evaluation of matrix effects based on adduct product ions revealed no significant interference from matrix components and acetonitrile in the ionization of both TXA and TXA-IS in plasma samples (Table 2), indicating that matrix-matching calibration was not required. Therefore, in this work, standards were prepared in the mobile phase, permitting to achieve high sensitivity determinations and decreasing the need for maintenance of the MS/MS detector. When matrix factor values were compared to those obtained previously using the *pseudo*-molecular ion of TXA [30], no significant differences were observed (|t|*_calculated_* < t*_tabulated_* (*p* = 0.05) = 2.23). Indeed, matrix factor values ≤ 105.4% were observed using the adduct ion-based strategy, reinforcing the promising application of this method for accurate TXA quantification.

**Figure 6:**
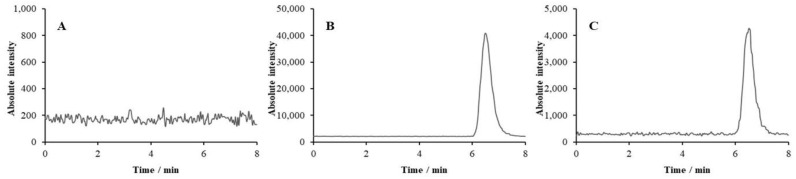
Representative chromatograms of a plasma extract from a patient before drug administration (**A**), spiked with 300 ng mL^−1^ TXA (**B**), and a plasma sample (35.7 ng mL^−1^ TXA in extract) collected from a patient undergoing scoliosis surgery (**C**).

Finally, the TXA quantification strategy based on the acetonitrile adduct was applied to the determination of TXA in plasma samples collected from five patients undergoing scoliosis surgery at two different time points: end of surgery and 24 h after surgery (Table 3). Chromatograms of a plasma sample collected from one of the patients, along with a blank plasma extract (collected before drug administration) and plasma spiked with TXA are presented in Figure 6. As the proposed method presented high precision and accuracy values complying with the requirements of established guidelines [37], and considering the reduced volume available of each plasma sample, the number of replicates per study sample was limited [38,39]. The obtained results (*C*_[M + ACN + H]_+) were compared with those determined using the *pseudo*-molecular ion of TXA (*C*_[M + H]_+) [30]. The statistical *t*-test was performed for each pair of experimental means and the |t|*_calculated_* values were lower than t*_tabulated_* (*p* = 0.05) = 4.30, in all cases. Hence, no statistically significant difference was found for the results obtained by the two methods.

In summary, the application of the adduct ion for quantification of TXA in plasma samples proved to be precise, accurate, sensitive, and provided results comparable to the values obtained using the *pseudo*-molecular ion. Moreover, the lower LOD and LOQ values indicate the suitability of adduct ions to quantify TXA at trace levels such as those occurring at extended pharmacokinetic studies (>48 h).

## 3. Materials and Methods

### 3.1. Chemicals and Solutions

All solutions were prepared using water from an Arium water purification system (resistivity > 18 MΩ cm, Sartorius, Göttingen, Germany). LC-MS grade chemicals were employed for analysis, namely acetonitrile (Merck, Darmstadt, Germany), formic acid (Merck, Darmstadt, Germany), and ammonium bicarbonate (Fluka, Buchs, Switzerland). Mixtures of acetonitrile, water, and aqueous ammonium bicarbonate (pH 7.4; 100 mM) were used to prepare the mobile phase, being filtered through 0.45 μm Millipore (Billerica, MA) HVHP filters and degassed for 15 min in an ultrasonic bath prior to use.

High purity (≥99%) analytical standards of tranexamic acid (TXA, Scheme 1) and the corresponding isotopically labeled internal standard ^13^*C*_2_,^15^*N*,*trans*-tranexamic acid (TXA-IS) were supplied by Toronto Research Chemicals (Toronto, Canada). Individual stock solutions of TXA and IS were prepared on a weight basis in water at a concentration of 1 mg mL^−1^ and stored at −20 °C. A working standard solution of TXA was prepared in water at 10 µg mL^−1^ to perform ionization studies. For quantification assays, working standard solutions of TXA (30, 60, 90, 150, 300, 450, 600 ng mL^−1^) were prepared in mobile phase by dilution of appropriate amounts of intermediate solutions at 20 and 1.5 μg mL^−1^. The internal standard TXA-IS was added at a final concentration of 300 ng mL^−1^.

### 3.2. Sample Preparation

The human plasma samples used in this work were obtained from a clinical study approved by the Ethics Committee for Health at Centro Hospitalar do Porto (process no. 2015.083(077-DEFI/072-CES)). The study was conducted after obtaining written informed consent from all participating subjects and in accordance with internationally recognized rules of good clinical practices. The TXA administration regimen to patients undergoing scoliosis surgery was carried out as described before [30]. The samples used for TXA quantification were collected at the end of surgery and 24 h later.

Sample preparation was performed as previously described [30]. Briefly, the proteins from human plasma samples containing TXA were precipitated by a mixture of 100 μL of each sample with 500 μL of acetonitrile containing 0.5% (*v*/*v*) of formic acid. After vigorous vortexing for 30 s, samples were centrifuged at 18,000× *g* for 6 min at 4 °C and supernatants were collected. TXA-IS was added at 300 ng mL^−1^ and finally, the sample extract was analyzed by ultra-high performance liquid chromatography coupled to triple quadrupole tandem mass spectrometry (UHPLC-MS/MS). Whenever necessary, sample extracts were diluted in the mobile phase before analysis.

### 3.3. Instrumentation and UHPLC-MS/MS Analysis

The quantity of TXA in plasma samples and standards was determined by UHPLC-MS/MS, according to the method previously described [30]. Briefly, analyzes were conducted in a Nexera X2 UHPLC system coupled to a triple quadrupole mass spectrometer detector LCMS-8040 with electrospray ionization (ESI) (Shimadzu Corporation, Kyoto, Japan). Data acquisition and processing was performed using LabSolutions software version 5.60 SP2 (Shimadzu Corporation).

Chromatographic separation was carried out in a BEH Amide column (50 × 2.1 mm, 1.7 μm; Waters, Milford, MA, USA), heated at 40 °C. The mobile phase composition consisted of a mixture of acetonitrile-aqueous ammonium bicarbonate (pH 7.4; 10 mM) (75:25, *v*/*v*). Elution was performed in isocratic mode, at a flow rate of 0.1 mL min^−1^, and each chromatographic run ended at 8.0 min. The injection volume was 0.2 μL.

The MS was operated in the positive ionization mode (ESI+) and data were acquired in SRM mode. The most abundant *m*/*z* transition 199.30 > 95.10 was employed for TXA-ACN protonated adduct quantification whereas the *m*/*z* transition 199.30 > 123.20 was used for identity confirmation. For comparison purposes, the precursor to product ion transitions employed previously [30] for TXA quantification and identification included the *m*/*z* transitions 158.25 > 95.15 and 158.25 > 123.20, respectively. The internal standard ^13^*C*_2_,^15^*N*,*trans*-TXA was monitored at *m*/*z* transitions 202.30 > 96.20 and 202.30 > 125.20, and at *m*/*z* transitions 161.25 > 96.15 and 161.25 > 125.20 [21,30].

### 3.4. Analytical Figures of Merit of Adduct Ion-Based Quantification Method

Linearity and working range were established by setting calibration curves in the range 30–600 ng mL^−1^. Each calibration standard was prepared and analyzed in triplicate, in three independent runs. Data were fitted to linear regression analysis to establish calibration curves for peak area ratio (TXA/TXA-IS) vs. the nominal concentration of TXA standard solutions. Back-calculated concentrations, i.e., the concentration values obtained for standard solutions when the corresponding analytical signal is interpolated in the equation of the calibration curve, were also determined [37].

The limit of detection (LOD) and limit of quantification (LOQ) values for TXA in plasma (*n* = 10) were determined as the minimum quantity of target analyte originating a signal-to-noise ratio of 3:1 and 10:1, respectively [40].

Method accuracy and precision were determined by intra- and inter-day analysis of one concentration level (300 ng mL^−1^) in the mobile phase and in blank plasma extracts spiked with TXA. Only one concentration level was selected instead of the three levels recommended by guidelines aiming at a comparison with the *pseudo*-molecular ion-based strategy for quantification of TXA, without the purpose of a thorough and full validation. The accuracy was reported as percent of the nominal concentration value and the precision was expressed by the coefficient of variation (CV). Six successive injections (*n* = 6) followed by interpolation in the calibration curve established in the same run were used to determine intra-day values. The data obtained from the replicate analysis of standards and spiked plasma extracts in three different runs (*n* = 3) were used to evaluate inter-day precision and accuracy.

Recoveries were determined by comparing the peak area of samples fortified with TXA and TXA-IS before sample treatment with the peak area of standards prepared at the same concentration (300 ng mL^−1^ of both TXA and TXA-IS). Furthermore, the matrix effect was investigated by the post-extraction spike method for the target analyte and IS at 300 ng mL^−1^, by calculating the ratio of the peak area in the presence of plasma matrix (blank plasma extracts spiked with TXA and TXA-IS) with the peak area in the absence of plasma matrix (solution of analytes in mobile phase) [37].

### 3.5. Computational Methods

In order to assess the relative stabilities of likely structures, density functional theory (DFT) calculations were performed with the Gaussian 16 package [41]. Geometry optimizations and vibration analysis were performed using the Zhao and Truhlar hybrid meta-GGA M06-2X functional [42] with the 6-31+G(d,p) basis set. Optimized structures were confirmed as energy minima, due to the absence of imaginary frequencies. The ESI process generates vapor phase ions that are analyzed within the mass spectrometer. Despite the mechanisms by which these ions are formed, the electrospray ionization occurs in a solvent environment [34,43], hence, for some calculations solvent was taken into account. For the calculations in the solvent environment, the SMD continuum solvation model [44] implemented in Gaussian16 was used with acetonitrile as solvent, using the default parameters. All the energies reported herein are corrected for the zero-point vibrational effects. Gibbs free energies at 298.15 K were evaluated on the geometries optimized in both phases. Thermal corrections to the Gibbs free energy were computed with Grimme quasi-harmonic corrections to entropy [45] for frequencies below 100 cm^−1^, due to the limitations of the harmonic oscillator approximation for low frequency modes [46]. Additional calculations on some systems were also performed at second-order Møller–Plesset MP2/6-311+G(2d,p) theory level.

## 4. Conclusions

The protonated acetonitrile adduct of TXA was observed as the most abundant ion formed under electrospray ionization when a mixture of acetonitrile-aqueous ammonium bicarbonate at pH 7.4 was used as mobile phase. The effect of source-dependent parameters, mobile phase composition and flow rate in the response observed for [M + H]^+^ and [M + ACN + H]+ was evaluated. The use of higher desolvation line temperatures and higher drying gas flow permitted to selectively enhance the signal of the [M + H]^+^ by favoring the desolvation of the adduct ion. Moreover, the reduction of the acetonitrile content in mobile phase and the use of reduced flow rates permitted to increase the ratio [M + H]^+^/[M + ACN + H]^+^ up to ca. 55%. Nevertheless, none of the tested conditions permitted to completely abolish the formation of the solvent adduct ions that continued to constitute the most abundant ion of the mass spectra.

DFT calculations provided some insights towards the possible structures for this acetonitrile protonated adduct of TXA. The adduct postulated herein as the energetically most stable is best described considering structure **7b** that is formed through the nucleophilic attack of the amino group of TXA into the electron-deficient carbon of the ethylnitrilium ion.

The formation and stability of solvent adducts was evaluated only for acetonitrile. The study of a single solvent can be regarded as a limitation of the present work, which can be expanded to other solvents in the future. The feasibility of applying the acetonitrile adduct for the quantification of TXA in plasma samples was addressed. The determined analytical figures of merit evidenced that the quantification of TXA based on the acetonitrile adduct presents high precision, accuracy, sensitivity and recovery values, and no significant matrix effects, making the method suitable for the intended application (trace analysis of TXA in plasma extracts). The higher signal intensities of the adduct ion permitted to achieve lower LOD and LOQ values in comparison to the *pseudo*-molecular ion-based quantification. Moreover, the analysis of TXA in clinical samples yielded results that were similar to the values obtained using the previous approach. Hence, the use of the acetonitrile adduct as the main quantifier ion instead of the protonated TXA molecule proved to be suitable for the determination of TXA in real plasma samples and improved the analytical sensitivity of the method developed.

## Figures and Tables

**Table 1:** Influence of mobile phase composition in TXA-ACN protonated adduct formation.

ACN Mobile Phase Composition (%, *v*/*v*)	[M + H]^+^/[M + ACN + H]^+^ Ratio (%)	
	Flow Injection	Chromatography
80	21.4 ± 1.5 ^I^	10.5 ± 0.1 ^II^
70	29.0 ± 1.9 ^III^	23.3 ± 1.4 ^IV^
60	39.4 ± 2.4 ^V^	29.5 ± 1.7 ^VI^
50	54.7 ± 4.2 ^VII^	33.8 ± 0.1 ^VIII^

^I–VIII^ Comparison between the mean ratio [M + H]^+^/[M + ACN + H]^+^ values obtained using flow injection analysis and chromatography. Different superscript numbers indicate that statistically significant differences were observed (*p* < 0.05).

**Table 2:** Figures of merit of the proposed UHPLC-MS/MS method for quantification of TXA based on the [M + ACN + H]^+^ adduct ion and comparison with the analytical parameters obtained using the *pseudo*-molecular [M + H]^+^ ion.

Parameters	Precursor ion [M + ACN + H]^+^ (This Work)	Precursor ion [M + H]^+^ [30]
Linear range (ng mL^−1^)	30–600 (180–3600 in plasma)	30–600 (180–3600 in plasma)
Calibration curve	y = 0.00291 (±0.00003) x + 0.026 (±0.009)	y = 0.00296 (±0.00002) x + 0.035 (±0.007)
Correlation coefficient (*r*^2^)	≥0.9983	≥0.9974
LOD (ng mL^−1^) ^a^	3	18
LOQ (ng mL^−1^) ^a^	6	36
Precision (CV, %) ^b^		
Intra-day	0.6	1.7
Inter-day	2.4	1.7
Accuracy (%) ^b^		
Intra-day	94.7	96.3
Inter-day	92.2	94.7
Recovery (%) ^c^		
TXA	98.8 ± 0.5 ^I^	96.3 ± 3.6 ^I^
TXA-IS	100.6 ± 1.0 ^II^	94.4 ± 1.0 ^III^
Matrix factor (%) ^d^		
TXA	105.4 ± 3.5 ^IV^	110.0 ± 7.6 ^IV^
TXA-IS	103.6 ± 6.9 ^V^	110.0 ± 6.3 ^V^
IS normalized matrix factor ^e^	102.0 ± 5.4 ^VI^	100.0 ± 1.4 ^VI^

LOD, limit of detection; LOQ, limit of quantification; CV, coefficient of variation; TXA, tranexamic acid; IS, internal standard, ^13^*C*_2_,^15^*N*,*trans*-tranexamic acid; ^a^ LOD and LOQ values in plasma samples; ^b^ Precision and accuracy estimated in plasma extracts spiked with 300 ng mL^−1^ TXA (corresponding to 1800 ng mL^−1^ in plasma); ^c^ Recovery (mean ± CV, *n* = 2) assessed by spiking plasma samples with 1800 ng mL^−1^ of TXA and IS before extraction and sample processing; ^d^ Matrix effect (mean ± CV, *n* = 6) assessed by the post-extraction addition method, at 300 ng mL^−1^ concentration level (in plasma extract); ^e^ IS normalized matrix factor calculated by division of the matrix factor of the analyte by the matrix factor of the IS; ^I–VI^ Comparison between the experimental means obtained for the different analytical parameters. The same superscript number indicates that no statistically significant difference was found for the results obtained by the two quantification methods (*p* < 0.05).

**Table 3:** Concentration of TXA (µg mL^−1^) in plasma samples collected from paediatric patients undergoing scoliosis surgery, determined using the *pseudo*-molecular [M + H]^+^ ion or the [M + ACN + H]^+^ adduct for quantification.

Plasma Samples ^a^	*C*_[M + ACN + H]_+ (This Work)	*C*_[M + H__]_+ [30]
End of surgery		
Patient 1	10.9 ± 0.1 ^I^	11.2 ± 0.1 ^I^
Patient 2	15.7 ± 0.1 ^II^	16.0 ± 0.1 ^II^
Patient 3	17.1 ± 0.5 ^III^	17.1 ± 0.2 ^III^
Patient 4	14.0 ± 0.3 ^IV^	14.2 ± 0.1 ^IV^
Patient 5	9.8 ± 0.1 ^V^	10.0 ± 0.1 ^V^
24 h after surgery		
Patient 1	0.213 ± 0.001 ^VI^	0.219 ± 0.002 ^VI^
Patient 2	0.226 ± 0.001 ^VII^	0.229 ± 0.001 ^VII^
Patient 3	0.325 ± 0.002 ^VIII^	0.332 ± 0.002 ^VIII^
Patient 4	0.214 ±0.004 ^IX^	0.226 ± 0.006 ^IX^
Patient 5	0.269 ± 0.004 ^X^	0.275 ± 0.001 ^X^

^a^ Each value corresponds to the mean ± standard deviation (*n* = 2). Prior to analysis by UHPLC-MS/MS, sample extracts collected at the end of surgery were diluted 20 times in mobile phase; ^I–X^ Comparison between the mean TXA concentration values determined in clinical plasma samples. The same superscript number indicates that no statistically significant difference was found for the results obtained by the two quantification methods (*p* < 0.05).

## Data Availability

Data is contained within the article and supplementary material.

## Supplementary Materials

The following are available online at https://www.mdpi.com/article/10.3390/ph14121205/s1, Figure S1. ESI-MS Scan spectra obtained for TXA in positive (I and III) and negative (II and IV) ionization modes, using acetonitrile-aqueous ammonium bicarbonate (pH 7.4; 10 mM) (80:20, *v*/*v*) (I and II) or acetonitrile-aqueous ammonium bicarbonate (pH 7.4; 10 mM) (50:50, *v*/*v*) (III and IV) as mobile phase; Figure S2. The ^1^H NMR of TXA were recorded on a BRUKER AVANCE III 400 MHz, 9.4 Tesla at 400.14 MHz, using tetramethylsilane as internal reference and a mixture of deuterated solvents composed by CD3CN/D2O (75:25, *v*/*v*) (A) in the absence or (B) presence of Na2CO3 additive.
