# Supplementary material for: Acetonitrile Adducts of Tranexamic Acid as Sensitive Ions for Quantification at Residue Levels in Human Plasma by UHPLC-MS/MS
Source: Pharmaceuticals (Basel). 2021 Nov 23;14(12):1205. doi: 10.3390/ph14121205 (PMC8708297; doi:10.3390/ph14121205)
Supplement: Supplementary file 1 [file pharmaceuticals-14-01205-s001.zip › pharmaceuticals-1436279-supplementary.pdf]

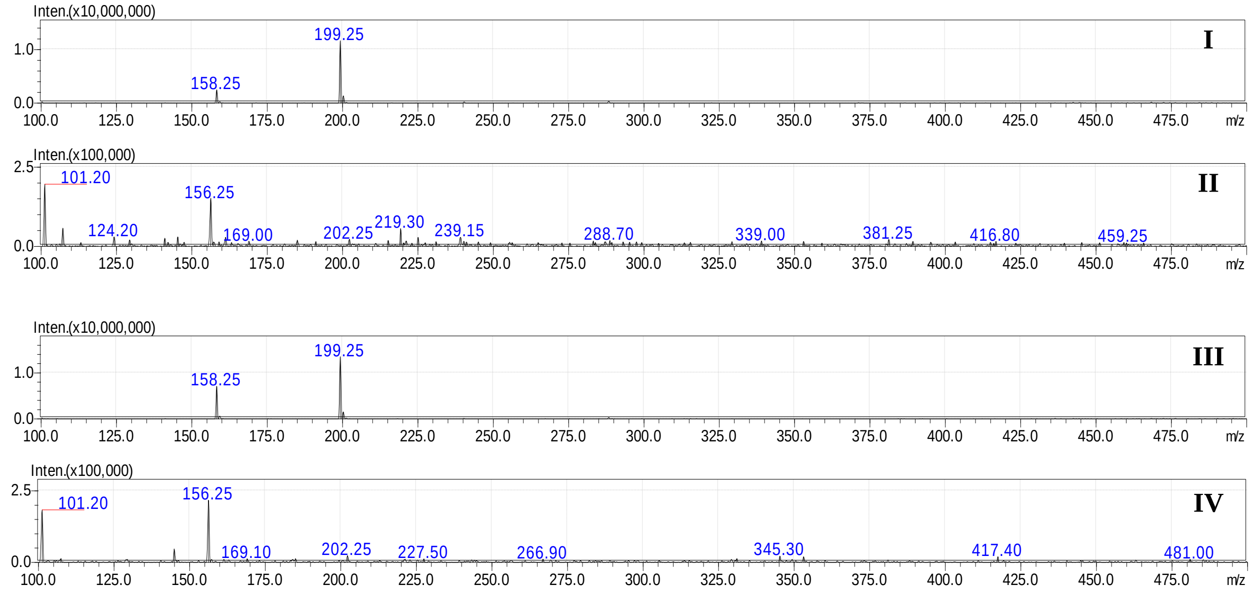

Figure S1. ESI-MS Scan spectra obtained for TXA in positive (I and III) and negative (II and IV) ionization modes, using acetonitrile-aqueous ammonium bicarbonate (pH 7.4; 10 mM) (80:20, v/v) (I and II) or acetonitrile-aqueous ammonium bicarbonate (pH 7.4; 10 mM) (50:50, v/v) (III and IV) as mobile phase.

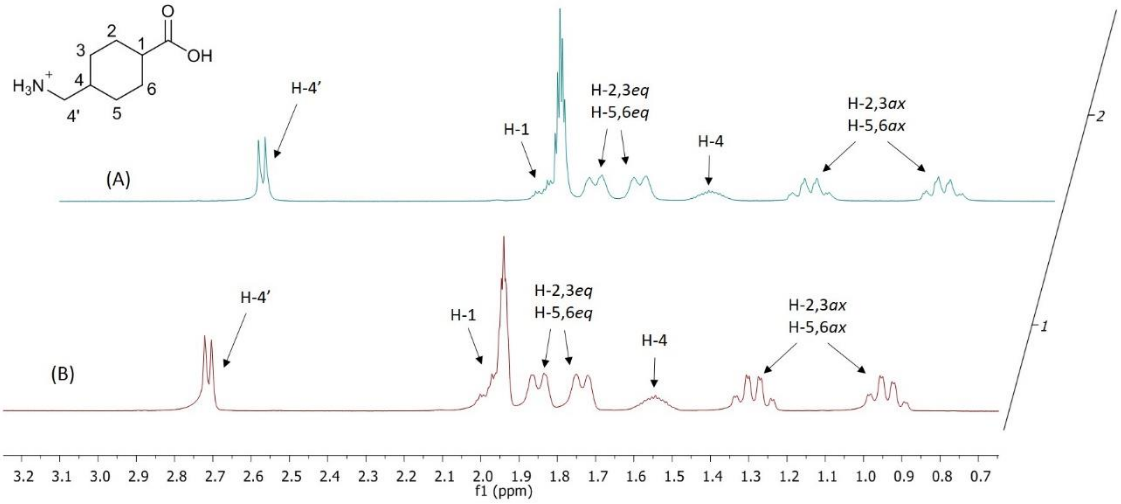

Figure S2. The ^{1}H NMR of TXA were recorded on a BRUKER AVANCE III 400 MHz, 9.4 Tesla at 400.14 MHz, using tetramethylsilane as internal reference and a mixture of deuterated solvents composed by CD_3CN/D_2O (75:25, v/v) (A) in the absence or (B) presence of Na_2CO_3 additive.
